# Nutritional Status Predicts Functional Recovery and Adverse Outcomes in Older Adults: A Prospective Cohort Study

**DOI:** 10.1002/jcsm.13819

**Published:** 2025-04-15

**Authors:** Ludiane Alves do Nascimento, Marlon Juliano Romero Aliberti, Natalia Golin, Erika Suíter, Christian Valle Morinaga, Thiago Junqueira Avelino Silva, Pedro Kallas Curiati

**Affiliations:** ^1^ Department of Nutrition Hospital Sírio‐Libanês Sao Paulo Brazil; ^2^ Geriatric Emergency Department Research Group (ProAGE) Hospital Sírio‐Libanês Sao Paulo Brazil; ^3^ Geriatric Center for Advanced Medicine Hospital Sírio‐Libanês Sao Paulo Brazil; ^4^ Laboratório de Investigação Médica em Envelhecimento (LIM‐66), Hospital das Clínicas University of Sao Paulo Medical School Sao Paulo Brazil; ^5^ Division of Geriatrics University of California San Francisco California USA

**Keywords:** clinical outcomes, emergency department, GLIM criteria, malnutrition, older adults

## Abstract

**Background:**

Despite the high prevalence of malnutrition in acutely ill older patients, nutritional status is rarely assessed in emergency departments (EDs), and the impact of nutritional risk screening on functional recovery is poorly understood. This study aimed to investigate the association between nutritional parameters and a range of outcomes in older patients admitted through the ED.

**Methods:**

A prospective cohort study was conducted at tertiary hospital, enrolling patients aged 65 years or older between November 2021 and April 2022. We collected data on various patient parameters, including demographics, clinical factors (Charlson Comorbidity Index [CCI], National Early Warning Score 2), nutritional status (Nutritional Risk Screening 2002; Global Leadership Initiative on Malnutrition criteria) and geriatric measures (Clinical Frailty Scale, Katz Index of Independence in Activities of Daily Living [ADL], Lawton and Brody Instrumental ADL, and PRO‐AGE vulnerability tool). The primary outcome was functional recovery, and secondary outcomes included nosocomial infection, prolonged length of stay (LoS), in‐hospital and postdischarge mortality, and hospital readmissions up to 6 months. Fine–Gray competing risks regression and multivariable logistic regressions were employed and adjusted for age, sex, education, CCI, functional status, LoS and initial allocation to intensive care.

**Results:**

A total of 780 patients (mean age 80 ± 9 years, predominantly male) were included, with 32.2% identified as at nutritional risk and 22.1% diagnosed with malnutrition. Patients with no nutritional risk had a higher significantly functional recovery up to 6 months (79% vs. 66%, sub‐HR = 1.28, 95%CI 1.04–1.57, *p* = 0.029), whereas nutritional risk was independently associated with in‐hospital (13% vs. 2%, OR = 4.24, 95%CI 1.53–11.74, *p* = 0.005) and postdischarge (14% vs. 4%, OR = 2.76, 95%CI 1.17–6.49, *p* = 0.02) mortality. Finally, malnutrition was independently associated with nosocomial infection (12% vs. 2%, OR = 5.43, 95%CI 2.56–11.5, *p* < 0.001), prolonged LoS (56% vs. 22%, OR = 2.79, 95%CI 1.84–4.22, *p* < 0.001) and postdischarge mortality (13% vs. 4%, OR = 2.76, 95%CI 1.36–5.61, *p* = 0.005).

**Conclusions:**

Nutritional parameters were significant predictors of functional recovery, nosocomial infection, prolonged LoS and mortality in older patients admitted through the ED. Early identification and interventions targeting nutritional deficiencies should be explored to improve outcomes in this vulnerable population.

## 1 Introduction

The ageing population presents a higher prevalence of chronic diseases, often overlapping with acute conditions, thus increasing the risk of malnutrition [1]. Malnutrition, a frequent condition among acutely ill older adults, negatively impacts clinical outcomes, affecting both the healthcare system and the patient [2]. Patients identified as nutritionally at risk or malnourished tend to utilize healthcare services more frequently and are more susceptible to adverse clinical outcomes. These outcomes include increased length of hospital stay (LoS), falls, functional decline, diminished quality of life, and heightened morbidity and mortality rates [3].

The emergency department (ED) often serves as the initial point of entry for hospital care, and early identification of nutritional risk in this setting allows for the timely implementation of interventions to mitigate these risks during hospitalization [4]. Several factors contribute to the elevated risk of malnutrition in older patients, encompassing nutrient malabsorption, inflammation and difficulties in chewing and swallowing [5]. The ageing process is also associated with a decline in muscle mass and strength, potentially leading to sarcopenia, dysphagia, frailty, immobility and cognitive impairment, all of them highly prevalent among older patients with malnutrition [6].

Identifying malnutrition upon hospital admission might contribute to risk stratification in acutely ill older patients, facilitating the development and implementation of improved care practices. Nutritional screening should be routinely performed after hospital admission as the initial step in identifying patients who are malnourished or at risk of malnutrition [7]. This screening process aims to direct patients towards a more comprehensive assessment when necessary. Various tools have been developed to screen hospitalized patients for nutritional risk, each with advantages and disadvantages [8, 9]. Although studies have compared different nutritional screening tools [10, 11, 12], there is no consensus on the optimal strategy.

Among the available instruments, the Nutritional Risk Screening 2002 (NRS‐2002), recommended by the European Society for Clinical Nutrition and Metabolism (ESPEN), stands out as a tool designed to identify nutritional risk in hospitalized patients [13]. In our hospital (Hospital Sírio‐Libanês, HSL), we apply the NRS‐2002 within the first 24 h of the patient's admission. If nutritional risk (NRS ≥ 3) is identified, we complement the assessment with the Global Leadership Initiative on Malnutrition (GLIM) to diagnose malnutrition [14].

To enhance the understanding of risk stratification in acutely ill older adults, this study investigated the association between nutritional parameters, such as nutritional risk and malnutrition, and various in‐hospital and postdischarge outcomes in older patients admitted through the ED. Particularly, we explored, for the first time, the impact of these factors on the recovery of baseline functionality up to 180 days postadmission.

## 2 Methods

### 2.1 Study Design

We conducted a prospective cohort study involving patients aged 65 years or older admitted through the ED of Hospital Sírio‐Libanês, a Brazilian tertiary hospital, between November 2021 and April 2022. This study derived from a larger cohort designed to explore the impact of geriatric vulnerability parameters on hospital and postdischarge outcomes in older patients admitted from the ED [15, 16, 17, 18, 19]. Patients were excluded from the study if they refused participation, could not be reached for assessment, required urgent procedures, were clinically unstable, could not communicate without assistance or lacked complete nutritional assessment data.

The study received approval from the Research Ethics Committee, and informed consent was obtained from all included patients or their legal representatives.

### 2.2 Data Collection

Trained researchers performed a standardized assessment with patients and their representatives in the ED. The assessment encompassed sociodemographic factors, medical history, comorbidities according to the Charlson Comorbidity Index (CCI), clinical severity according to National Early Warning Score (NEWS) 2, nutritional status (including Nutritional Risk Screening 2002 [NRS‐202] and Global Leadership Initiative on Malnutrition [GLIM] criteria) and geriatric assessment (including frailty according to the Clinical Frailty Scale [CFS], baseline functionality according to Katz Index of Independence in Activities of Daily Living [ADL], Lawton and Brody Instrumental ADL, Barthel Index of ADL and vulnerability according to PRO‐AGE score). Data on length of hospital stay, adverse events and in‐hospital mortality were extracted from electronic medical records. Patients or their representatives were contacted via telephone at 30, 90 and 180 days after hospital admission to assess ED revisits, hospital readmissions and mortality during these periods.

### 2.3 Nutritional Assessment

The NRS‐2002 was applied within the first 24 h of admission, and if nutritional risk was identified (NRS ≥ 3), the assessment was supplemented with the GLIM criteria for malnutrition diagnosis. The assessment of muscle mass, necessary for the application of the GLIM criteria, was performed using a bioelectrical impedance analysis (BIA) and, if it was contraindicated or not available, with anthropometric measurements [20]. This practice is supported by ESPEN's guidance for assessment of muscle mass phenotypic criterion with a level of agreement of 92%, noting that anthropometry is generally less sensitive than appropriately implemented imaging or bioelectrical impedance methods [21]

The BIA assessment was carried out with a portable device compatible with bedside testing [20]. Muscle mass was also evaluated using the Appendicular Skeletal Muscle Mass Index, adjusted for the individual's height [22]. These data were used to determine normal muscle mass or reduced muscle mass when < 7.0 kg/m^2^ for men and < 5.7 kg/m^2^ for women [14]. In cases where BIA was contraindicated, calf circumference was measured, using the normality cutoff points validated for the Brazilian population by Barbosa‐Silva et al. [23]: greater than 34 cm for men and greater than 33 cm for women.

Our nutritional assessment team's protocols are described elsewhere [20].

### 2.4 Outcomes

The primary outcome was time to functional recovery, defined as the first occurrence of a Katz Index score equal to or greater than the baseline score assessed upon hospital admission.

Secondary outcomes included nosocomial infection, prolonged LoS, in‐hospital and postdischarge mortality and hospital readmissions up to 6 months.

### 2.5 Statistical Analysis

Analyses were performed using Stata Version 17 (StataCorp, College Station, TX). All statistical tests were two‐tailed, with an alpha error of 0.05. Numerical variables were reported as means and standard deviations or medians and interquartile ranges (IQR) depending on their distribution, which was assessed by visual inspection of histograms, calculation of skewness and kurtosis coefficients and D'Agostino–Pearson test for normality. Categorical variables were described as absolute counts and proportions. Comparisons between distributions of numerical variables used Student's t‐test or ANOVA for normal distributions and Wilcoxon or Kruskal–Wallis tests for nonnormal distributions. Categorical variables were compared using the chi‐square test or Fisher's exact test when appropriate.

Fine–Gray competing risks regression was used to explore the association between nutritional status and time to functional recovery, with death as a competing risk, excluding totally dependent patients (Katz score of 0) at hospital admission. This model was chosen due to its ability to handle time‐to‐event data with competing risks, providing a more realistic view of conditional probabilities. Cumulative incidence curves illustrated the association between nutritional status categories assessed at baseline and ADL functional recovery withing 180 days of admission.

Multivariable logistic regressions were used to investigate the association between nutritional status and the other clinical outcomes. Analyses were adjusted for age, sex, education, comorbidities, functionality and initial allocation to intensive care unit (ICU) at admission.

**FIGURE 1:**
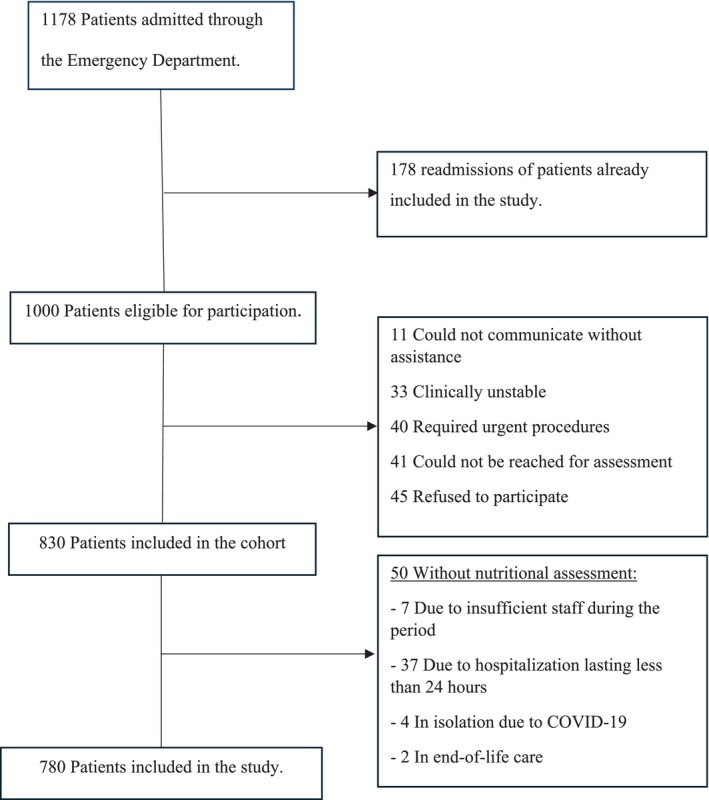
Flowchart of the study participants.

Complete case analysis was used, as subjects with incomplete baseline data were excluded from the study population (Figure 1).

## 3 Results

A total of 780 patients were included in the study (Figure 1), with a mean age of 80 (± 9) years and a male predominance (53%). Nutritional risk (NRS‐2002 ≥ 3) was identified in 251 (32.2%) patients, of whom 172 (22.1% of the total sample) were classified as malnourished according to the GLIM criteria (Figure 1). These patients were older (83 ± 8 vs. 78 ± 8 years), had a lower mean body mass index (22.2 vs. 26.3 kg/m^2^) and reported recent weight loss more frequently (60% vs. 9%) than those without nutritional risk (Table 1). They also exhibited higher comorbidity rates, particularly anaemia, dementia and Parkinson's disease, along with increased medication use and higher scores on clinical severity (NEWS2), frailty (CFS) and geriatric vulnerability (PRO‐AGE) [17, 24, 25] scales (Table 1). Furthermore, malnourished patients experienced longer hospital stays (9 vs. 5 days), were more often transferred to the ICU due to clinical deterioration (18% vs. 7%), had higher rates of hospital‐acquired infections (12% vs. 2%) and pressure injuries (3% vs. 0%) and demonstrated increased mortality rates both during (8% vs. 2%) and after (13% vs. 4%) hospitalization (Table 1).

**TABLE 1:** Characteristics of the study population stratified according to nutritional status.

	Total	No nutritional risk	Nutritional risk without malnutrition	Nutritional risk and malnutrition	*p* value
	*N* = 780	*N* = 529	*N* = 79	*N* = 172	
Sociodemographic factors					
Age (years), mean (SD)	80 (± 9)	78 (± 8)	82 (± 9)	83 (± 9)	< 0.001
Female, *n* (%)	364 (47)	248 (47)	30 (38)	86 (50)	0.20
White, *n* (%)	715 (92)	491 (93)	69 (87)	155 (90)	0.13
Education (years), Median (SD)	14 (± 5)	14 (± 5)	13 (± 5)	14 (± 5)	0.057
Comorbidities					
Anaemia, *n* (%)	56 (7)	27 (5)	9 (11)	20 (12)	0.005
Dementia, *n* (%)	149 (19)	78 (15)	18 (23)	53 (31)	< 0.001
Depression, *n* (%)	141 (18)	93 (18)	12 (15)	36 (21)	0.48
Diabetes mellitus, *n* (%)	236 (30)	155 (29)	29 (37)	52 (30)	0.41
Parkinson's disease, *n* (%)	41 (5)	19 (4)	2 (3)	20 (12)	< 0.001
Hypertension, *n* (%)	404 (52)	280 (53)	42 (53)	82 (48)	0.47
Heart failure, *n* (%)	124 (16)	74 (14)	13 (16)	37 (22)	0.063
Cancer, *n* (%)	201 (26)	129 (24)	21 (27)	51 (30)	0.38
Clinical variables					
NEWS, Median (IQR)	1 (0–3)	1 (0–3)	2 (0–3)	2 (1–4)	< 0.001
Charlson Comorbidity Index, median (IQR)	2 (1–3)	0 (1–3)	2 (1–3)	3 (1–3)	0.019
Polypharmacy, *n* (%)	621 (80)	403 (76)	69 (87)	149 (87)	0.003
Regular physical activity, *n* (%)	319 (41)	239 (45)	33 (42)	47 (27)	< 0.001
Frailty (CFS ≥ 5), *n* (%)	216 (28)	98 (19)	35 (44)	83 (48)	< 0.001
PRO‐AGE score, median (IQR)	2 (1–4)	2 (1–3)	3 (1–5)	4 (2–5)	< 0.001
Kartz scale, median (IQR)	0 (0–4)	0 (0–1)	1 (0–5)	3 (0–6)	< 0.001
Barthel index, median (IQR)	95 (60–100)	100 (85–100)	90 (30–100)	70 (33–100)	< 0.001
Memory complaints, *n* (%)	303 (39)	175 (33)	38 (48)	90 (52)	< 0.001
Delirium, *n* (%)	157 (20)	76 (14)	20 (25)	61 (35)	< 0.001
Anthropometric parameters					
Weight (kg), median (IQR)	72 (62–82)	74 (65–85)	76 (65–85)	62 (54–72)	< 0.001
BMI (kg/m^2^), median (IQR)	26 (23–29)	26 (24–29)	27 (24–30)	22 (20–25)	< 0.001
Weight loss last 3 months, *n* (%)	178 (23)	46 (9)	28 (35)	104 (60)	< 0.001
Outcomes					
Nosocomial infection, *n* (%)	35 (4)	12 (2)	2 (3)	21 (12)	< 0.001
Pressure ulcers, *n* (%)	7 (1)	0 (0)	1 (1)	6 (3)	< 0.001
Length of stay (days), median (IQR)	6 (3–10)	5 (3–8)	7 (4–12)	9 (6–17)	< 0.001
Prolonged LoS, *n* (%)	239 (31)	116 (22)	27 (34)	96 (56)	< 0.001
Unplanned admission to ICU, *n* (%)	74 (9)	35 (7)	8 (10)	31 (18)	< 0.001
ED revisits up to 6 months, *n* (%)	168 (24)	103 (21)	25 (38)	40 (26)	0.007
Rehospitalization up to 6 months, *n* (%)	168 (24)	103 (21)	25 (38)	40 (26)	0.007
Functional recovery at 6 months, *n* (%)	586 (75)	419 (79)	54 (68)	113 (66)	< 0.001
In‐hospital mortality, *n* (%)	35 (4)	11 (2)	10 (13)	14 (8)	< 0.001
Postdischarge mortality, *n* (%)	55 (7)	22 (4)	11 (14)	22 (13)	< 0.001

Abbreviations: BMI, body mass index; CFS, Clinical Frailty Scale; ED, emergency department; ICU, intensive care unit; IQR, interquartile range; LoS, length of stay; NEWS, National Early Warning Score; SD, standard deviation.

**FIGURE 2:**
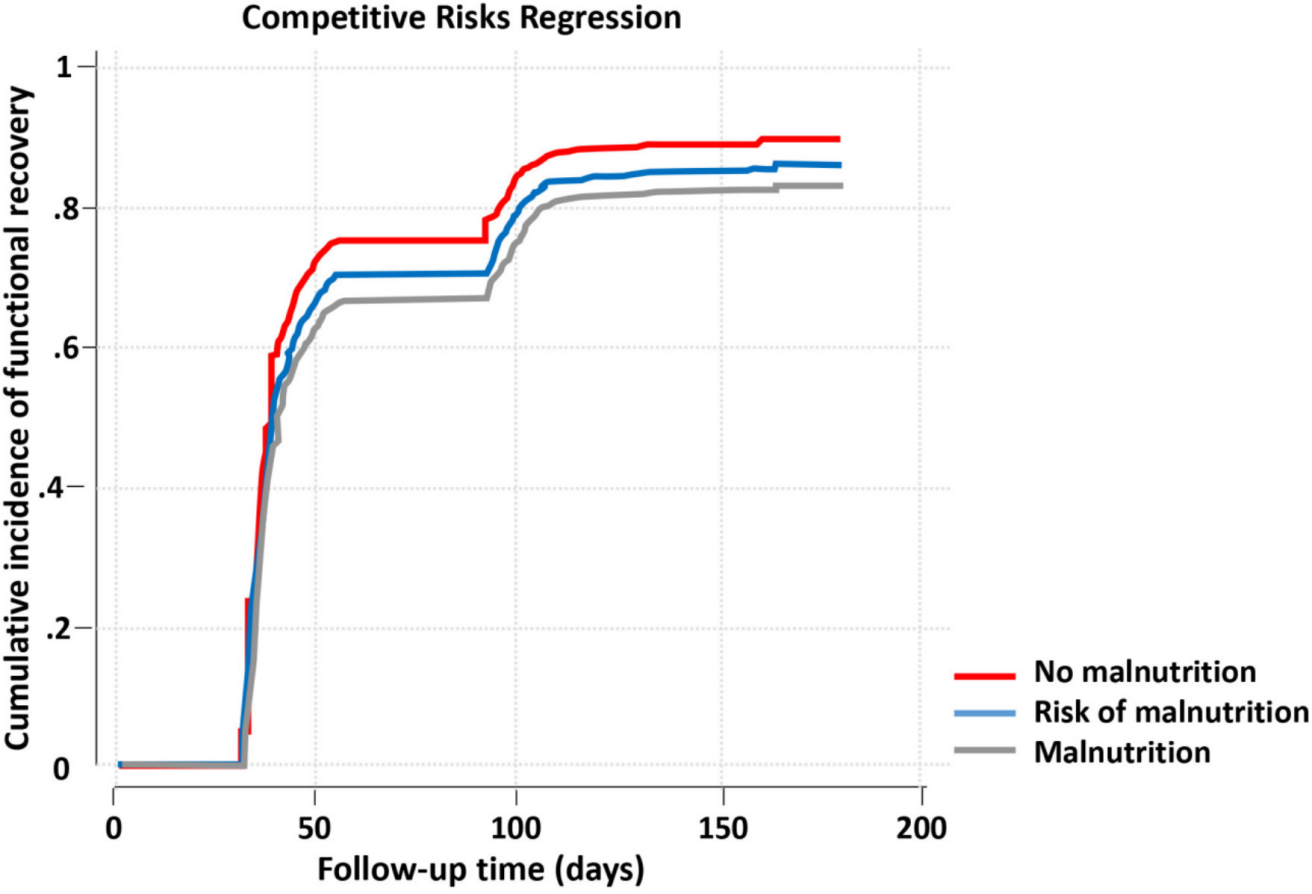
Time‐to‐functional‐recovery curves.

The time‐to‐functional‐recovery curves revealed a faster recovery of baseline functionality in patients without nutritional risk within the first 30 days, followed by a slower recovery that still favoured those without nutritional risk (Figure 2). In the analysis of the association between nutritional status and time to functional recovery, conducted using Fine–Gray regression and adjusted for age, sex, education, comorbidities (CCI), functionality (Katz Index of Independence in ADL and Lawton Instrumental ADL Scale), prolonged hospitalization and initial allocation to ICU, patients without nutritional risk had a 28% greater recovery of baseline functionality at the end of 6 months follow‐up period as compared to malnourished patients (Table 2). Sixty‐two patients were considered lost to follow‐up in these analyses due to missing functional status data at 180 days.

**TABLE 2:** Fine–Gray competing risks for the association between nutritional status and functional recovery up to 6 months.^a^

	sHR	95% CI	*p* value	sRRa^b^	95% CI	*p* value
Malnutrition	Ref.	Ref.	Ref.	Ref.	Ref.	Ref.
Nutritional risk without malnutrition	1.22	0.85–1.75	0.278	1.10	0.80–1.53	0.555
No nutritional risk	1.85	1.50–2.29	< 0.001	1.28	1.04–1.57	0.019

Abbreviations: CI 95%, 95% Confidence Interval; Ref., reference; sHR, subhazard ratio; sRRa, adjusted subhazard ratio.

^a^ Totally dependent patients (Katz score of 0) at hospital admission were excluded.

^b^ Analyses were adjusted for age, sex, education, comorbidities (CCI), functionality (Katz and Lawton scales) and initial allocation to intensive care unit (ICU) at admission.

Logistic regression analyses, adjusted for age, sex, education, comorbidities, functionality and ICU admission, showed that patients with malnutrition had nearly threefold higher odds of a prolonged hospital stay (*p* < 0.001), were more than five times as likely to develop a hospital‐acquired infection (*p* < 0.001) and had nearly triple the odds of postdischarge mortality (*p* = 0.005), compared with those who had no nutritional risk (Table 3). Malnutrition was not significantly associated with hospital readmission, adverse events or in‐hospital mortality. In contrast, patients who were at nutritional risk but not malnourished had over fourfold higher adjusted odds of in‐hospital mortality (*p* = 0.005) and nearly threefold higher adjusted odds of postdischarge mortality (*p* = 0.020) compared to those with no nutritional risk (Table 3). No significant associations were observed for hospital‐acquired infection, prolonged hospital stay or hospital readmission in this group. Also, there was no statistically significant difference in mortality risk among subsets of patients with nutritional risk (those with and those without malnutrition).

**TABLE 3:** Logistic regression analyses between nutritional status and secondary outcomes.

	Nutritional risk without malnutrition^a^				Malnutrition^a^			
	OR (95% CI)	*p* value	aOR^b^ (95% CI)	*p* value	OR (95% CI)	*p* value	aOR^b^ (95% CI)	*p* value
Nosocomial infection	1.11 (0.24–5.09)	0.884	0.94 (0.20–4.39)	0.94	5.99 (2.88–12.4)	< 0.001	5.43 (2.56–11.5)	< 0.001
Prolonged LoS	1.85 (1.11–3.07)	0.018	1.3 (0.73–2.30)	0.37	4,5 (3.12–6.48)	< 0.001	2.79 (1.84–4.22)	< 0.001
In‐hospital Mortality	6.82 (2.80–16.66)	< 0.001	4.24 (1.53–11.74)	0.005	4.17 (1.86–9.38)	0.001	1.91 (0.74–4.90)	0.18
Postdischarge mortality	4.28 (1.97–9.26)	< 0.001	2.76 (1.17–6.49)	0.02	3.65 (1.96–6.78)	< 0.001	2.76 (1.36–5.61)	0.005
Rehospitalization	1.51 (0.87–2.60)	0.14	1.34 (0.75–2.38)	0.32	1.56 (1.06–2.30)	0.024	1.08 (0.70–1.68)	0.71

Abbreviations: 95% CI, 95% confidence interval; aOR, adjusted odds ratio; LoS, length of stay; OR, odds ratio.

^a^ Reference: no nutritional risk.

^b^ Analyses were adjusted for age, sex, education, comorbidities (CCI), functionality (Katz and Lawton scales) and initial allocation to intensive care unit (ICU) at admission.

## 4 Discussion

In this prospective cohort study of acutely ill older adults admitted through the ED, we found that nutritional risk and malnutrition were independently associated with a reduced likelihood of functional recovery and an increased risk of adverse in‐hospital and postdischarge outcomes, including nosocomial infection, prolonged length of stay (LoS) and mortality. These findings are innovative and expand upon previous research demonstrating the detrimental impact of malnutrition on various health outcomes in hospitalized older adults [26, 27, 28, 29].

Our study builds upon previous research by focusing on a vulnerable population of acutely ill older adults admitted through the ED, a setting where nutritional assessments are not routinely performed. By identifying nutritional risk and malnutrition as significant predictors of adverse outcomes in this population, our findings underscore the importance of integrating nutritional assessments into routine ED care for older adults. This could facilitate early identification of at‐risk patients and enable timely interventions to improve their clinical trajectory.

Although there exists an overlap between nutritional status and frailty [30, 31], a study conducted on a cohort of multimorbid patients revealed that only one third of frail individuals exhibited sarcopenia [32]. Research from Korea has further highlighted an interaction between these parameters in predicting mortality and long‐term hospitalization [33]. Indeed, malnutrition is a widespread issue among hospitalized patients, affecting 20%–50% of adults [34] and 35%–65% of older adults [35], and can be regarded as a public health concern. Despite its prevalence, it remains underdiagnosed [36].

Furthermore, our study goes beyond previous research by examining the impact of nutritional status on functional recovery, a patient‐centred outcome crucial for maintaining independence and quality of life in older adults. The association between nutritional risk, malnutrition and reduced functional recovery highlights the need for interventions that address acute medical issues and prioritize nutritional support and rehabilitation to optimize long‐term outcomes.

Our study has limitations. The observational design precludes causal inferences, and the single‐centre setting may limit the generalizability of our findings. Also, the 6‐month inclusion and follow‐up periods might be vulnerable to potential seasonal bias in emergency care needs and outcomes among older patients. Additionally, the assessment of reduced food intake relied on patient or caregiver reports rather than objective measures, although this reflects the practical limitations of clinical practice in many hospitals. Although we did not stratify patients based on nutritional interventions, all patients identified with nutritional risk received in‐hospital nutritional intervention and guidance for continued care after discharge. Despite these limitations, the real word ED setting, the prospective design, the two‐step nutritional assessment, devised to optimize time and resources, and the use of a patient‐centred primary outcome make our findings appealing and practice changing. Future studies should investigate the impact of specific nutritional interventions on functional recovery and other clinical outcomes in this population.

## 5 Conclusion

In conclusion, our study demonstrates the significant association between nutritional risk, malnutrition and adverse outcomes in acutely ill older adults admitted through the ED. These findings suggest early nutritional assessments and interventions in this vulnerable population might have a role in improving both short‐ and long‐term clinical outcomes, including functional recovery. Addressing malnutrition in the ED can potentially enhance the quality of care and outcomes for older adults, ultimately promoting their well‐being and independence.

## Conflicts of Interest

The authors declare no conflicts of interest.
